# Multidrug resistance in bacteria associated with leafy greens and soil in urban agriculture systems

**DOI:** 10.3389/fpls.2025.1664284

**Published:** 2025-09-22

**Authors:** Erin Harrelson, Qingyue Zeng, Mairui Gao, Magaly Toro, Ryan A. Blaustein

**Affiliations:** ^1^ Department of Nutrition and Food Science, University of Maryland, College Park, MD, United States; ^2^ Joint Institute of Food Safety and Applied Nutrition, University of Maryland, College Park, MD, United States

**Keywords:** urban agriculture, whole genome sequencing, antimicrobial resistance, environmental resistome, plasmids, *Pseudomonas*

## Abstract

Urban farms and community gardens support local food production, though these agroecosystems can contain emerging environmental contaminants that may contribute to the dissemination of antimicrobial resistance (AMR). Our previous research enumerated AMR bacteria associated with leafy vegetable production environments in the greater Washington, D.C. area, identifying >100 isolates with multidrug-resistant (MDR) phenotypes. Here, we performed whole genome sequencing analysis of 87 of these strains recovered from leafy greens (n=29), root zone soil (n=42), and bulk soil (n=16) to comprehensively characterize their MDR genotypes, including taxonomy and any encoded ARGs, stress response genes, and mobile genetic elements (MGEs; e.g., plasmids, phages, conjugative elements). The MDR isolates spanned 4 phyla and 14 genera, with the majority identified as *Pseudomonas* (n = 29), *Serratia* (n = 22), *Providencia* (n = 11), and *Bacillus* (n = 11). Most of the ARGs were linked to multidrug efflux, while other abundant ARG classes reflected resistance to beta-lactams and tetracyclines. While the genotypes were often conserved within respective species and even genera, the observed phenotypes within taxonomic groups slightly varied, suggesting the potential roles of uncharacterized genetic elements in MDR function. Moreover, all of the MDR isolates encoded at least one gene annotated as a MGE, and there were 19 distinct ARGs located within 5,000 bp upstream or downstream of these sequences, suggesting potential implications for mobilization. Overall, our results indicate that the MDR bacteria in urban agriculture systems, including on fresh produce, are dominated by general soil-associated taxa that carry diverse ARGs and MGEs.

## Introduction

With the world population rapidly growing, addressing food and nutritional insecurity remains a critical issue. Urban farms and community gardens provide numerous benefits to society, from strengthening local food production to engaging communities and increasing green space in cities. However, contamination of urban soils by pollutants that may accumulate due to dense human activities, along with potential damages to soil fertility (e.g., depleted organic matter, compaction), presents difficulties for establishing resilient food systems (Yang and Zhang, 2015; Delgado-Baquerizo et al., 2021). Biological contaminants such as human pathogens and bacteria with antimicrobial resistance (AMR) can become elevated in urban soils as well (Nguyen et al., 2019; Delgado-Baquerizo et al., 2021). Ensuring safe and sustainable urban food production may require innovative strategies to promote both soil and crop health.

AMR poses significant challenges in the United States, contributing to an estimated 2.8 million infections and over 35,000 deaths annually (Centers for Disease Control and Prevention (U.S.), 2019; Sutton and Ashley, 2024). Agriculture has been implicated as a major contributor to the spread of AMR and the emergence of multidrug-resistant (MDR) bacteria. Antibiotic use in livestock can lead to high levels of antimicrobial residues and AMR genes (ARGs) in animal manures, which can persist even after composting or other treatment processes (Gurmessa et al., 2020; Wang et al., 2023). In addition, chemical contaminants common in crop landscapes, such as pesticides and heavy metals, are known to exert selective pressures that may influence the selection of AMR bacteria (Wang et al., 2021; Fu et al., 2023; Schoen et al., 2024). Bacteria harboring ARGs can colonize crop surfaces and internal tissues, including those of fresh produce (Zhang et al., 2019), underscoring the relevance of One-Health frameworks for understanding food safety risks in agricultural systems (Franklin et al., 2024). Nevertheless, little is known about the spectrum of AMR bacteria and ARGs that may persist throughout urban agriculture environments.

Fresh produce is an emerging yet understudied reservoir for AMR and MDR microorganisms. Fruits and vegetables naturally harbor complex microbial communities, ranging from 10^3^–10^8^ bacteria per gram (Abadias et al., 2008; Fiedler et al., 2017), some of which carry intrinsic or acquired ARGs (Kiplimo et al., 2025). Under harsh environmental conditions (e.g., chemical exposure, nutrient limitation, temperature flux), ARGs and stress response genes involved in virulence, biocide resistance, and heavy metal resistance may be co-selected (Naditz et al., 2019; Diaconu et al., 2021). These adaptive processes are mediated by gene transfers involving mobile genetic elements (MGEs), which have been documented in a variety of fresh produce, including leafy greens, carrots, tomatoes, and herbs (Rahman et al., 2021; Chen et al., 2023). Moreover, while the extent of risks posed by foodborne AMR bacteria and ARGs remains unclear, foodborne illnesses still impact nearly 10 million people in the U.S. each year (Scallan et al., 2011; Walter et al., 2025). Given that ARGs can enhance the persistence of foodborne pathogens and complicate treatment options, understanding the types of AMR bacteria and ARGs present in urban food systems, along with the potential for transmission, has important implications for food safety and public health.

Organic soil amendments are essential for restoring soil health for urban farming, though their effects on AMR bacteria are reportedly inconsistent (Liu et al., 2024). Our recent work demonstrated that organic inputs at urban farms can reduce the proportions of ampicillin- and tetracycline-resistant bacteria in amended soils compared to the native soils, perhaps reflecting microbial community shifts that mitigate or dilute resident AMR populations (Zeng et al., 2024). However, we also observed a high incidence of MDR among AMR isolates recovered from both leafy green vegetables and soils, suggesting the presence of broad-spectrum resistance mechanisms and/or co-selection of AMR genes (ARGs). Building on these findings, the present study used whole genome sequencing (WGS) analysis to test the hypothesis that MDR in urban agriculture environments and on preharvest produce is primarily associated with persistent, soil-dwelling strains (i.e., not foodborne pathogens per se), although the ARGs they carry may be linked to MGEs and pose potential risks for mobilization.

## Methods

### Sampling and MDR bacteria isolation

Our previous microbiological field survey of urban and peri-urban farms (n=5 sites) and community gardens (n=2 sites) across greater Washington, D.C. (Zeng et al., 2024) generated a unique culture collection of MDR bacteria isolates. In brief, leafy green vegetables (n=92 plants of kale [*Brassica oleracea* and *Brassica napus*], cabbage [*Brassica rapa* subsp. *pekinensis*], lettuce [*Lactuca sativa*], or chard [*Beta vulgaris*] varieties) and the plots or raised beds in which they were grown were sampled using aseptic technique. Leaf tissue (LT) and root zone soil (RZS) were collected by combining up to four leaves per plant or three soil cores within a 20cm distance from the plant stem, respectively, into sterile Nasco Whirl-pak bags (Pleasant Prairie, WI, USA). In addition, bulk soil (BS) samples representing ‘native’ or unamended soil at each site was collected from several locations along the site perimeter (i.e., >10m apart), again by combining three soil cores taken at each sampling position. The LT (n=92), RZS (n=92), and BS (n=39) samples were transferred to the laboratory for enrichment isolation of AMR bacteria. Total bacteria resistant to ampicillin (Amp) or tetracycline (Tet) were enumerated from the respective samples on trypticase soy agar (TSA) (MP Biomedicals, Solon, OH, USA) augmented with ampicillin sodium salt (32 μg ml^-1^) or tetracycline hydrochloride (16 μg ml^-1^) following incubation at 37 °C for 24h. Isolates with unique colony morphologies were maintained in 25% glycerol at -80 °C and, later, screened for additional AMR phenotypes. We employed a replica plating assay in which isolates were grown on Mueller Hinton agar augmented with Amp (32 μg ml^-1^), Tet (16 μg ml^-1^), azithromycin (Azi; 32 μg ml^-1^), ciprofloxacin (Cip; 4 μg ml^-1^), chloramphenicol (Chl; 32 μg ml^-1^), cefotaxime (Cef; 1 μg ml^-1^), or gentamycin (Gen; 16 μg ml^-1^) (i.e., National Antimicrobial Resistance Monitoring System concentration breakpoints (https://www.cdc.gov/narms/antibiotics-tested.html). The isolates with phenotypes for resistance to three or more of the seven antibiotic classes tested were considered MDR. Thus, using the culture collection obtained in our previous study (Zeng et al., 2024), the present study focused on characterizing the AMR genotypes and related features of these isolates (n=105) through WGS and bioinformatics analysis.

### Sequencing and bioinformatics analysis

Glycerol stocks of the MDR isolates were streaked onto TSA, and pure cultures were grown in trypticase soy broth (TSB), each incubated at 37 °C for 24h. The biomass was pelleted via centrifugation at 10,000 x *g* for 1min. DNA was extracted from the pellets using the Quick-DNA™ Fungal/Bacterial Miniprep Kit (Zymo Research, Irvine, California), and concentrations were quantified with a Qubit 4 fluorimeter (Invitrogen, Waltham, Massachusetts). Negative control DNA extractions (n=2) were run in parallel and yielded negligible DNA concentrations. WGS libraries were prepared using the Illumina DNA Prep kit with UD Indexes (Illumina, San Diego, CA). Paired-end sequencing (2x150 cycles) was performed targeting 75x coverage. The whole genome sequencing of these isolates was performed on an Illumina NextSeq1000 at the Joint Institute for Food Safety and Applied Nutrition.

Sequenced reads were preprocessed using Trimmomatic v0.4 (Bolger et al., 2014) with parameters ‘ILLUMINACLIP TruSeq3-PE.fa:2:15:10:1 LEADING:20 TRAILING:20 SLIDINGWINDOW:4:20 MINLEN:36’ and assembled using Spades v4.0.0 (Prjibelski et al., 2020) with default parameters. Assembled genomes were run through CheckM v1.2.3 (Parks et al., 2015) for quality control, and those with predicted completeness greater than 95% and contamination less than 5% were considered as ‘high-quality’ and used in downstream analysis (n=87).

The high-quality genomes were given taxonomic assignments to the species level with GTDB-Tk v2.4.0 (Chaumeil et al., 2022). For assemblies that did not receive a species assignment (i.e., only classified to the genus level, thus representing putatively novel species), dRep v3.1.0 (Olm et al., 2017) on Kbase (Arkin et al., 2018) was used to identify redundancies and determine the number of unique species based on an ANI threshold of 0.95. All genomes were then queried for genes encoding proteins with homology to AMR and virulence-associated features using the core AMRFinderPlus v3.12.8 (database 2024-07-22.1; Feldgarden et al., 2021) and, for complementary profiling, the Comprehensive Antibiotic Resistance Database Resistance Gene Identifier (CARD-RGI; https://card.mcmaster.ca/analyze/rgi) tool (Alcock et al., 2023) with default parameters. The mobileOG (Brown et al., 2022) database mobileOG-db-beatrix-1.6 with Diamond (Buchfink et al., 2015) v2.1.11.165 was used to further classify genes associated with MGEs (e.g., plasmid, integrase, transposase), omitting hits with ‘ACLAME only.’ The top hits were selected based on the highest bitscore per query.

### Statistical analysis

All data visualizations were prepared using R v. 4.4.1 using packages ggplot2 (Wickham, 2016), Plotly (Sievert, 2020), Complex Heatmap (Gu, 2022), and Circlize (Gu et al., 2014). ANOVA was applied to compare isolate genome size across sample types (i.e., LT, RZS, BS). Chi-squared tests were used to compare the numbers of MDR isolates representing different genera, AMR phenotypes, and AMR genotypes (i.e., gene presence/absence) as a factor of sample type as well.

## Results

### Taxonomic classification of multidrug-resistant isolates

Our WGS approach yielded high-quality genome assemblies for 87 MDR isolates recovered from urban farms and community gardens, with 29 isolates derived from 20 leaf tissue (LT) samples, 42 isolates from 32 root zone soil (RZS) samples, and 16 isolates from 12 bulk soil (BS) samples.

The genome size was greater in isolates recovered from both RZS (5,690 ± 1,092 kbp) and BS (5,554 ± 788 kbp) than in LT (4,931 ± 530 kbp) (ANOVA *P* < 0.05; mean ± standard deviation), which was in line with trends for several of the taxa identified differing across sample types.

**Figure 1:**
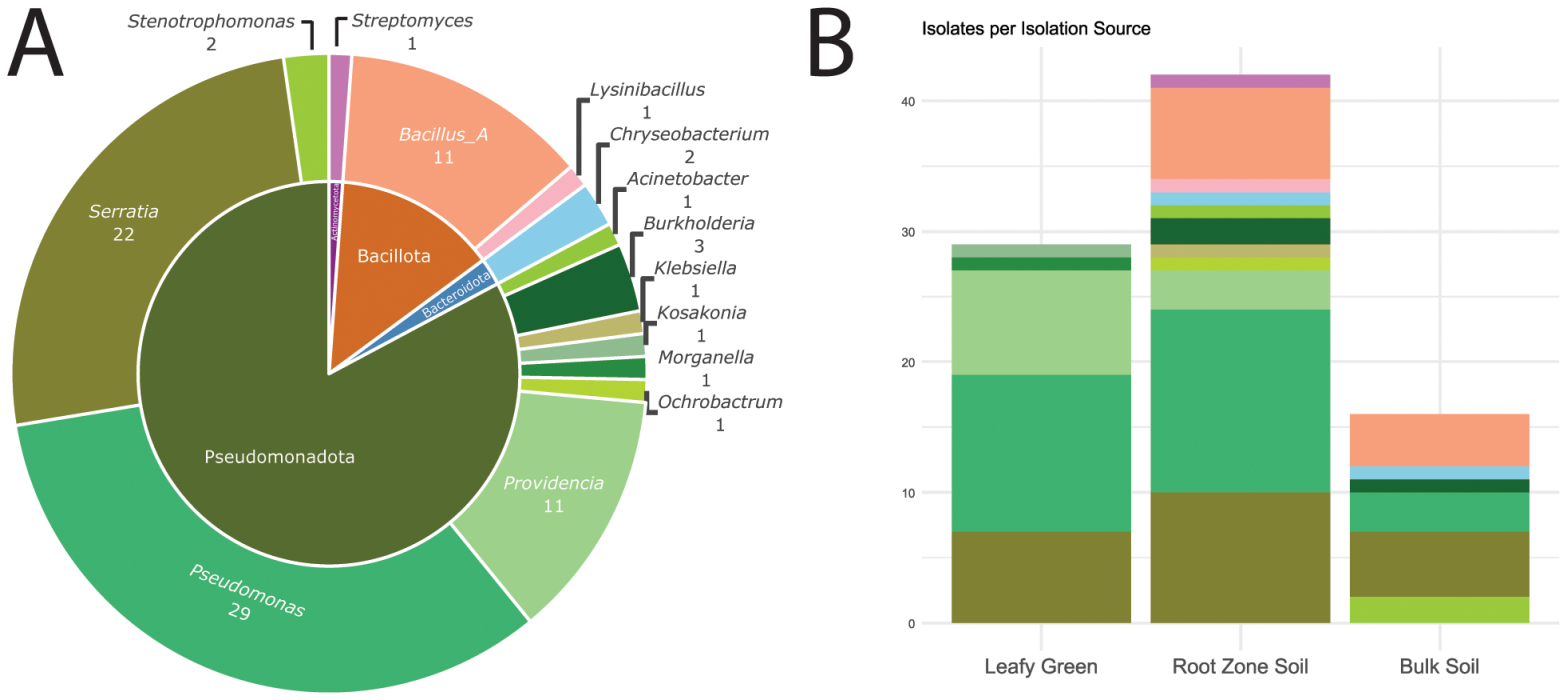
Taxa identified among the MDR isolates recovered from urban farms and community gardens (n = 87). **(A)** Distributions of phyla (inner circle) and genera (outer circle) assigned. **(B)** Number of MDR isolates derived from each source type. The color corresponds to the genus colors in **(A)**.

The majority of the MDR isolates were members of the phylum Pseudomonadota (n = 73 isolates, 84%), including 16 different species of *Pseudomonas* (n = 29 isolates), 5 species of *Serratia* (n= 22), and 4 species of *Providencia* (n = 11) (**Figure 1A**). Within Pseudomonadota, there were also 2 species of *Burkholderia* (n = 3), 2 species of *Stenotrophomonas* (n = 2), *Klebsiella planticola* (n = 1), *Acinetobacter gyllenbergii* (n = 1), *Morganella morganii* (n = 1), *Ochrobactrum cystisi* (n = 1), and *Kosakonia cowanii* (n = 1). The second most prominent phylum among the MDR strains was Bacillota (n = 12 isolates, 14%). Specifically, there were 6 species of *Bacillus* (n = 11) and *Lysinibacillus capsici* (n = 1). Among the four major genera represented by more than 10 MDR isolates each, the respective proportions of *Pseudomonas* and *Serratia* did not differ by sample type (*P* > 0.05 for each). However, *Providencia* was disproportionately associated with the leafy greens (*P* = 0.009; 27.6% of LT, 7.1% of RZ, and 0.0% of BS isolates), while *Bacillus* was only recovered from soils (*P* = 0.030; 0.0% of LT, 16.7% of RZ, and 25.0% of BS isolates) (**Figure 1B**; **Supplementary Table 1**).

Additional phyla represented among the MDR strains were Actinomycetota and Bacteroidota (**Figure 1A**), including one novel species of unclassified *Streptomyces* (n = 1 isolate) and 2 novel species of unclassified *Chryseobacterium*, as distinguished by ANI (n = 1 isolate of each; **Supplementary Figure 1A**). The additional novel species’ identified included an unclassified *Providencia* (n = 2 isolates; **Supplementary Figure 1B**) and an unclassified *Pseudomonas* (n = 1 isolate). In total, there were 44 distinct species identified among the 87 isolates (i.e., 14 in LT, 30 in RZ, and 13 in BS, with some overlapping), with the most abundant being *Serratia ureilytica* (n = 10), *Pseudomonas fulva* (n = 6), and *Pseudomonas manganoxydans* (n = 5). These MDR strains largely appeared to reflect general soil-associated taxa and, to our knowledge, no known foodborne pathogens were identified (Scallan et al., 2011; Walter et al., 2025).

### AMR phenotypes and genotypes among isolates

**Figure 2:**
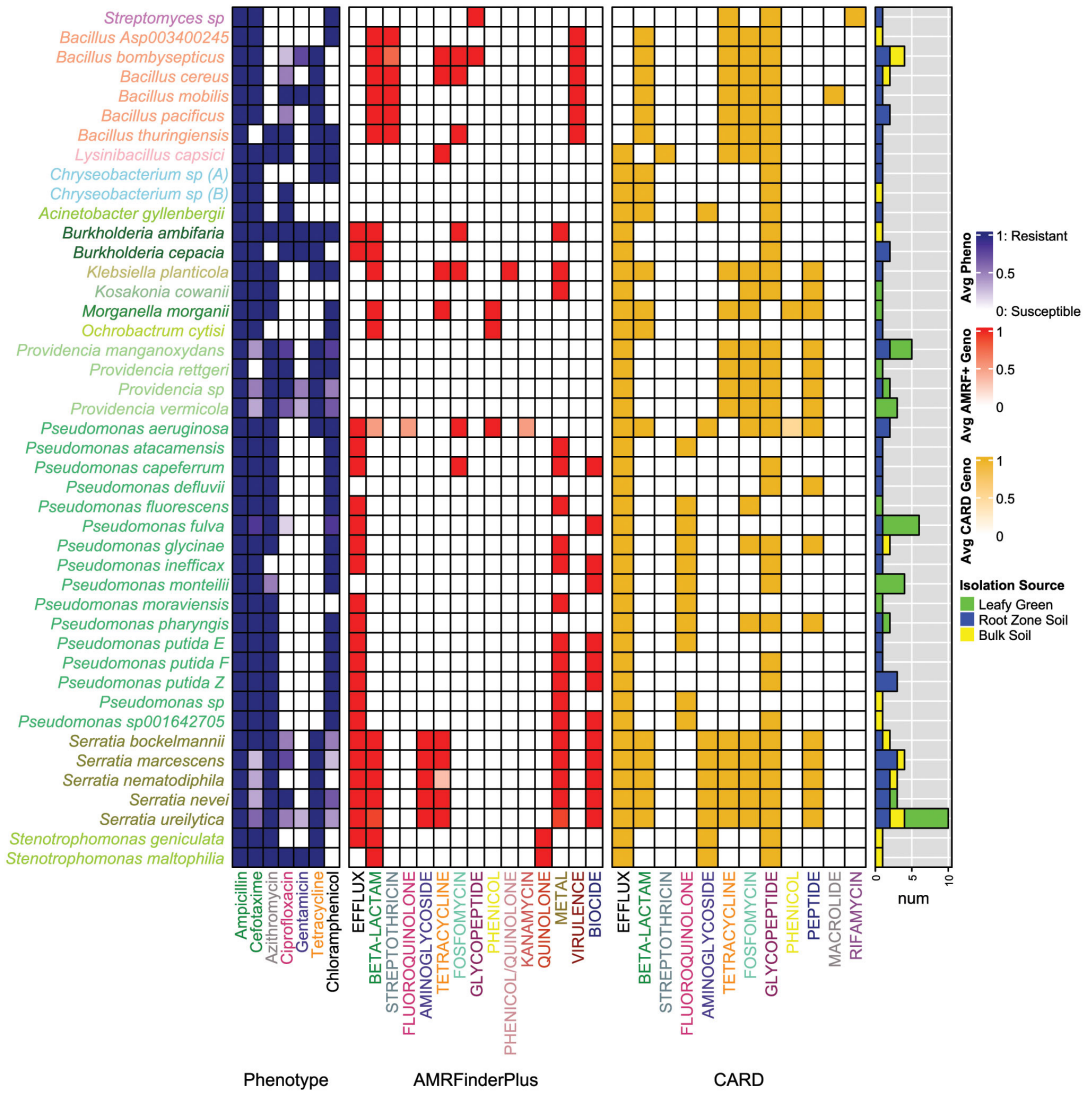
AMR phenotypes and genotypes for MDR bacteria species. Heat colors reflect average values for all isolates within the respective species. Taxa label colors correspond to the genus classifications in **Figure 1A**. The heatmap from left to right displays: (1) AMR phenotypes, (2) AMR genotypes as determined by AMRFinderPlus, and (3) AMR genotypes as determined by CARD. Label colors for the listed phenotypes and genotypes correspond by the antibiotic classes represented. The right panel shows the number of isolates per species derived from the respective isolation source types (i.e., leafy green, amended soil, bulk soil).

The MDR isolates’ phenotypes were previously determined against 7 different antibiotics (Zeng et al., 2024) and are shown in **Figure 2**. Those sequenced were all resistant to Amp (100%) and many to Tet (60.9%), which is in line with their initial recovery on media augmented with these antibiotics (i.e., n = 40 and 47 isolates from Amp and Tet media, respectively). A high proportion of these isolates were also resistant to Cef (77%), a third-generation cephalosporin. Moreover, 77.0%, 59.8%, 39.1%, and 14.9% of the isolates were resistant to Azi, Chl, Cip, and Gen, respectively. MDR patterns followed trends based on sample types. For example, those recovered from leafy greens were more often resistant to Azi (0.042; 93.1% of LT, 69.1% of RZ, and 68.8% of BS isolates) and Chl (*P* = 0.014; 75.9% of LT, 59.5% of RZ, and 31.3% of BS isolates) compared to those recovered from soils. Alternatively, MDR isolates from BS were more often resistant to Gen than those from RZS or LT (*P* = 0.020; 10.3% of LT, 9.5% of RZ, and 37.5% of BS isolates). These trends may reflect resistance mechanisms associated with the elevated proportions of *Providencia* and *Bacillus* among the MDR isolates recovered from leafy greens and soil, respectively, as previously described. MDR isolate phenotypes for Amp, Cef, Cip, and Tet were not different by sample type (*P* > 0.05 for each; **Supplementary Table 1**).

**Figure 3:**
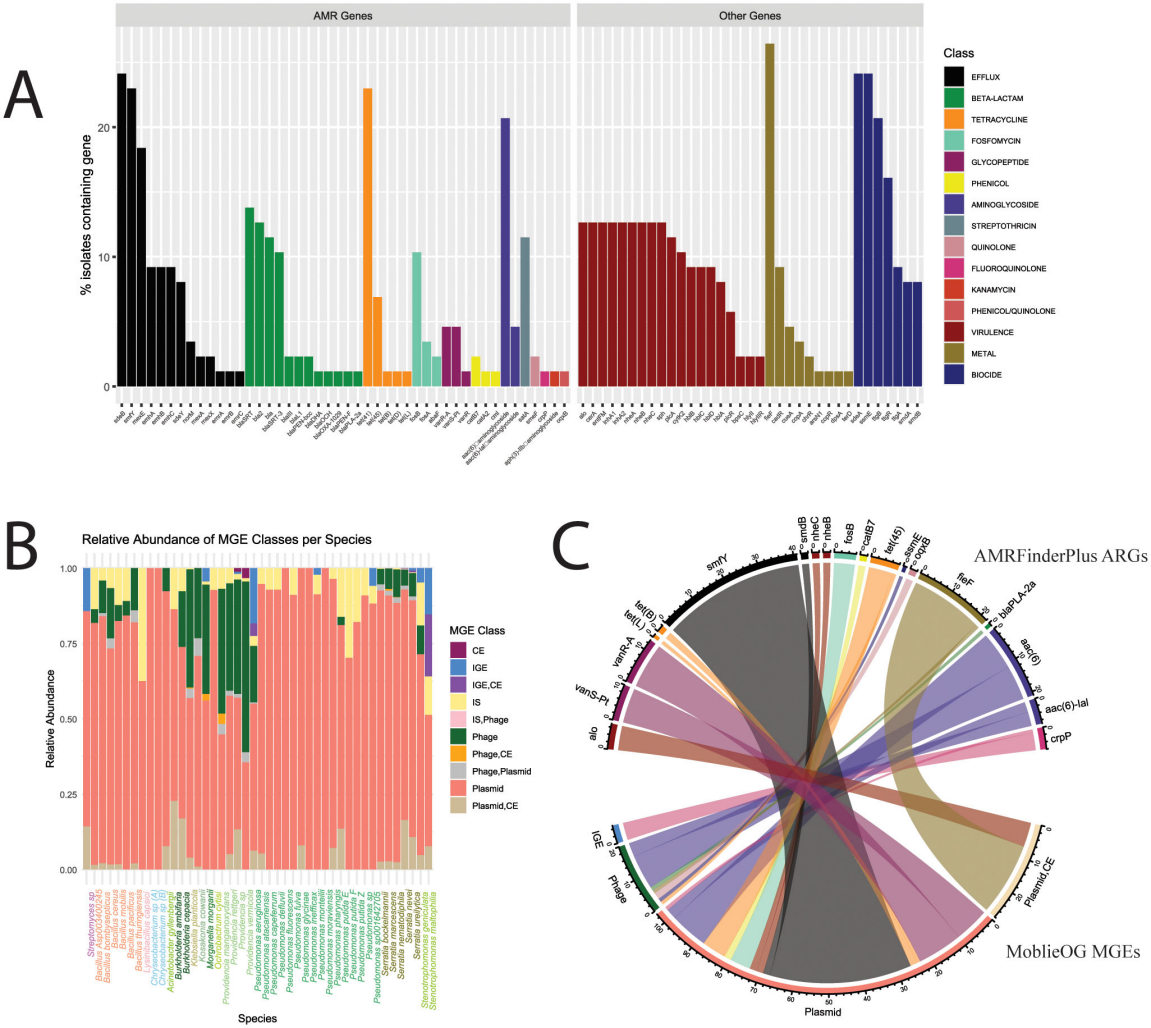
AMR genes identified by AMRFinderPlus and potential associations with MGEs. **(A)** Percent of isolates carrying each gene, with the colors corresponding to antibiotic class. **(B)** Relative abundance of MGE classes identified among genomes for each species. **(C)** Counts for occurrences of MGEs and AMR genes found within 5,000 bp on the same contig.

To explore AMR genotypes, we used two AMR profiling tools for complementary characterization. AMRFinderPlus and CARD-RGI identified 46 and 158 distinct ARGs, respectively (**Figure 3A**; **Supplementary Figure 2A**). In addition, AMRFinderPlus identified 7, 9, and 19 genetic elements associated with biocide resistance, heavy metal tolerance, and virulence, respectively. Notably, the virulence genes identified were only associated with the *Bacillus* genus.

Observations of phenotype and genotype (**Supplementary Table 2**) were made at the genus- and species-levels (**Figure 2**). *Pseudomonas* (n=29 isolates) exhibited phenotypes of resistance to Azi (89.7% resistant) and Chl (93% resistant), with infrequent resistance to Cip (3.4% resistant), Tet (6.9% resistant), and Gen (0% resistant). According to AMRFinderPlus, *Pseudomonas* AMR genotypes were the most diverse relative to the other groups and encoded efflux pumps (i.e., *mexE, emhC, emhB, emhA, mexX, mexA*), heavy metal tolerance (i.e., *cueA, copA, copR, arsN1, cadR, chrR*), and biocide resistance (i.e., *ttgA, ttgB, ttgT*). In contrast to AMRFinderPlus, CARD detected the *adeF* efflux gene in 93% of *Pseudomonas* isolates and vancomycin resistance genes in 10 species. The most represented species in the collection was *P. fulva* (n = 6), which exhibited slight phenotypic variation, though consistent genotypes (e.g., *mexE*-AMRFinderPlus; *adeF* and *Abau_AbaQ*-CARD). Moreover, *P. aeruginosa* carried the highest number of ARGs among all *Pseudomonas*, despite being represented by only two isolates (i.e., 8 genes associated with resistance to 6 antibiotic classes were detected by AMRFinderPlus, and 12 genes linked to resistance to 7 antibiotic classes were identified by CARD).

Efflux genes appeared to be conserved among other Pseudomonadota as well. For example, the MDR *Serratia* isolates were all resistant to Amp (100%), Azi (100%), and Tet (100%), while showing less frequent resistance to Cip (54.5%), Chl (36.3%), Cef (50%), and Gen (13.4%). Among the *Serratia* isolates, AMRFinderPlus identified genetic elements involved in multidrug efflux (*smfY, sdeB, Ysde*), tetracycline efflux (*tet(41)*), and enzymatic resistance to beta-lactams (*blaSRT, blaSRT-3*) and aminoglycosides (*aac(6)-lal, aac(6)*). Metal and biocide genes were also frequently identified among *Serratia*, with 13 out of 16 total stress-related genes detected in the study encoded by at least one *Serratia* strain, a frequency higher than in other genera. The most abundant of these genes were the cation-efflux transporter *fieF* (64%) and the biocide efflux pumps *ssmE* (59%) and *sdeA* (55%). CARD also identified similar beta-lactam, tetracycline, and aminoglycoside genes, along with an additional 7 efflux genes, as well as peptide, fosfomycin, and glycopeptide resistance genes. The *Serratia* isolates exhibited different phenotypes across species, despite having relatively similar AMR genotypes. Within *Serratia nevei*, UF-187 and UF-268 encoded the same ARGs, though the former was resistant to twomore antibiotics (i.e., Cef and Chl), while UF-274 encoded an additional efflux gene (*sdeY*), yet was sensitive to Cef. *S. ureilytica* was the most prominent species represented among all isolates and carried an average of 4.9 ± 0.7 AMRFinderPlus ARGs and 3.2 ± 0.6 stress-associated genes. Despite the consistency in *S. ureilytica* genotypes, phenotypic resistance varied across four antibiotics (i.e., Cip-50%, Chl-40%, Cef-60%, and Gen-30%), with some isolates demonstrating resistance to 5 to 7 antibiotics, while others were resistant to only three.

*Providencia* (n=11) was, on average, resistant to approximately 5 of the antibiotics tested (i.e., Az-100%, Tet-100%, Cip-81.8%, Chl-72.7%, Cef-36.3%, Gen-18.2%) (**Figure 2**). However, AMRFinderPlus did not identify any AMR genes in *Providencia*, perhaps reflecting database biases against understudied taxa. Nevertheless, several ARG hits were identified by CARD, including a variety of genes associated with antibiotic efflux (i.e., *adeF, KpnH, qacG, CRP, rsmA*), as well as resistance to vancomycin (i.e., van cluster genes), tetracycline (*tet(59)*), phosphonic acid (*FosA8*), and peptide antibiotics (*ArnT*). Similarly, *Acinetobacter gyllenbergii* were resistant to Amp, Cip, and Cef and *Kosakonia cowanii* resistant to Amp, Azi, and Cef, AMR genotypes were not detected by AMRFinderPlus for either species. However, both were found to contain 8 and 20 AMR genes as characterized by the CARD database. Thus, using multiple databases for AMR characterization was essential to identify potential genotypes that may help to explain the observed resistance phenotypes.

Moreover, another species within Pseudomonadota, *Burkholderia ambifaria*, was resistant to all antibiotics tested, and *Burkholderia cepacia* was resistant to all but Azi and Cip. According to AMRFinderPlus, the two *B. cepacia* encoded efflux gene *norM* and beta-lactam gene *blaPEN-bcc*. The *B. ambifaria* isolate carried both genes as well as *abaF*, a Fosfomycin resistance gene. CARD further identified efflux genes *adeF*, *amrA*, *amrB*, *ceoB*, and *qacG* for both isolates, as well as an additional van cluster gene for *B. cepacia.* The other less common Pseudomonadota isolates were (1) *K. planticola* with resistance to all but Cip and Gen antibiotics, encoding *fosA*, *oqxB*, *tet(B)*, *blaPLA-2a*, and *fieF;* (2) *M. morganii* with resistance to Amp, Azi, Chl, and Cef, encoding *catA2*, *blaDHA, and tet(D);* and (3) *O. cytisi* with resistance to Amp, Chl, and Cef, encoding *cml* and *blaOCH*. Thus, AMR genotypes aligned with phenotypes for isolates of the less commonly dominant MDR species as well.

In contrast to the Pseudomonadota, members of MDR Bacillota showed variable phenotypes. The *Bacillus* isolates (n=11) were all resistant to Amp (100%), mostly resistant to Cef (91%) and Tet (91%), though less consistently resistant to Cip (45%), Gen (36%), Chl (18%), and Azi (9%) (**Figure 2**). All *Bacillus* isolates had at least one beta-lactam gene (e.g., *bla2, bla, blaIII*) and streptothricin gene (e.g., *satA*), while half had a fosfomycin resistance gene (e.g., *fosB*) identified by AMRFinderPlus. Glycopeptide genes (*vanS-Pt* and *vanR-A*) were observed as well in *B. bombysepticus*. CARD analysis indicated that 4/6 of the species carried *tet(45)* and 6/6 had *tetB(P)*, which may play roles in the high frequency of Tet resistance observed within *Bacillus*. Moreover, *L. capsici* was resistant to 6 out of 7 antibiotics (i.e., Amp, Azi, Cip, Chl, Gen, and Tet). While only one tetracycline resistance gene was identified by AMRFinderPlus (*tet(L)*), a more diverse set of 7 total AMR genes was found for this isolate when using CARD. Of these genes, Vancomycin (i.e., *vanW* in the *vanI* cluster, *vanY* in the *vanM* cluster, and *vanT* in the *vanG* cluster), drug efflux (i.e., *qacJ*), Streptothricin (i.e., *msr(G)*), Fosfomycin (i.e., *FosBx1*), and Tetracycline (i.e., *tet(45)*) drug classes were represented.

The two novel *Chryseobacterium* species showed different phenotypes, both displaying resistance to Amp and Cef, with one further resistant to Chl and Tet, while the other was only additionally resistant to Cip. While AMRFinderPlus did not identify any AMR genes in these isolates, CARD identified genes with homology to *adeF* and *qacG* associated with efflux, *IND-7* (i.e., a beta-lactamase), and *vanT* in the *vanG* cluster (i.e., confers glycopeptide resistance). Moreover, the novel *Streptomyces* species was resistant to Amp, Chl, and Cef and carried a gene associated with glycopeptide resistance (*vanR*), as distinguished by AMRFinderPlus. CARD identified additional *Streptomyces-*specific rifamycin (*HelR, Sven_rox*) and tetracycline genes (*otr(A)*), as well as several glycopeptide resistance genes (*vanH* in the *vanO cluster, vanI*, and *vanX* in the *vanI* cluster).

Several AMR genes appeared to be significantly enriched by sample type. As identified by AMRFinderPlus, *blaSRT-3* (i.e., identified only in *Serratia* isolates) was the only gene more frequently present in MDR isolates from leafy greens (*P* = 0.043; 20.7% of LT, 2.4% of RZ, and 12.5% of BS isolates), while three alternative beta-lactamase genes (*bla*, *bla2*, and *blaL1*) and *smeF* were only found in those recovered from soil (*P* < 0.05 for each; all were 0.0% in LT and ranged from 12.5% to 25.0% in RZ and BS). Several additional genes identified by CARD were slightly yet significantly enriched among the LT isolates (i.e., *Abau_AbaQ, Kpne_KpnH*, *tet(59)*). In contrast, there were 13 genes associated with efflux and resistance to aminoglycosides and beta-lactams that were found more frequently among the soil-derived isolates (e.g., *smeA*, *smeD*, *APH(9)-Ic, BcI, BcIII*) (*P* < 0.05 for each) (**Supplementary Table 1**).

Overall, there was a diverse set of AMR phenotypes and genotypes among the MDR strains recovered from the leafy vegetables and soil at the urban farms and community gardens. Incorporating multiple AMR profiling tools enabled the broader characterization of ARGs. As described above, there were 46 and 158 unique genes identified by AMRFinderPlus and CARD, respectively. Among the genome assemblies for all MDR isolates (n=87), these AMR genetic elements had a total of 244 hits (i.e., AMRFinderPlus ARGs, excluding stress and virulence) and 1,038 hits, respectively. To evaluate consistencies across the two databases, we investigated sequence position matches within 100 bp on the same contig. There were 190 of the same hits annotated as ARGs by both AMRFinderPlus and CARD (**Supplementary Table 3**). Genes encoding proteins associated with AMR classes of aminoglycoside, tetracycline, beta-lactam, fosfomycin, glycopeptide, and phenicol antibiotics were consistently identified within this overlapping set, albeit with some alternative gene names (e.g., *blaDHA* and *DHA-4* both as beta-lactamases). An exception to this was the multiple matches to the *adeF* efflux pump classified by CARD, which matched multiple times with 4 other efflux genes in AMRFinderPlus (i.e., *ttgB*, *sdeB*, *sdeY*, and *emhB)*.

### Mobile genetic elements associated with ARGs

MGEs, including plasmids, conjugative elements (CE), insertion sequences (IS), phages, and interactive elements (IGE), were identified using mobileOG (**Figure 3B**). Plasmids were the most abundant class of MGE and the only one with gene signatures present in every MDR isolate genome. To link MGEs with ARGs via associations as local neighborhood genes, we screened for genes within 5,000 bp (i.e., within approximately 5 genes upstream or downstream) on the same contig (**Figure 3C**, **Supplementary Table 4**). Regarding ARGs identified with AMRFinderPlus hits, 19 ARGs were found to be located within this distance to MGEs, including (i) the multidrug efflux pump *smfY* associated with plasmid sequences (n = 41 hits), (ii) the heavy metal tolerance gene *fieF* associated with ‘plasmid, CE’ (i.e., indicating conjugative plasmid; n = 21 hits), (iii) aminoglycoside resistance genes *aac(6)* and *aa(6)-lal* associated with phage (n=14 hits) and plasmid (n=14 hits) sequences, and (iv) tetracycline genes *(tet(L)*, *tet(B)*, and *tet(45)*) primarily linked to plasmids (**Figure 3C**). A similar frequency of putative MGE associations with CARD-identified ARGs was characterized as well (**Supplementary Table 5**; **Supplementary Figure 2B**).

## Discussion

Urban agriculture has gained popularity for its positive impacts on communities and sustainability. However, heterogeneity in urban landscapes is associated with dynamic environmental conditions that shape local soil and crop microbiomes (Nugent and Allison, 2022). That is, farming conditions can vary widely from site to site depending on soil properties, depletions in organic matter, the presence of urban contaminants (e.g., heavy metals, anthropogenic chemicals, emissions), and differences in resources that are available for crop management (e.g., soil amendments, irrigation water sources). Under variable sublethal environmental stresses, AMR bacteria can become selected and ARGs may propogate through horizontal gene transfers (Samreen et al., 2021). Although AMR and emergent multidrug resistance (MDR) are increasingly recognized risks for food safety (Doyle, 2015), understanding specific implications within urban agriculture is not well known. The present study aimed to understand the types of MDR bacteria and the diversity of ARGs and MGEs present throughout urban agriculture preharvest environments, from urban soils to leafy green produce.

The MDR isolates comprised 44 species within 14 genera, primarily members of Pseudomonadota (e.g., *Pseudomonas, Serratia*, and *Providencia)* and Bacillota (e.g., *Bacillus*). Notably, there were trends for sample-type differences, as *Pseudomonas* and *Serratia* were universal, while *Providencia* appeared to associate with the plant leaves and roots, and *Bacillus* and more diverse MDR taxa were only recovered from the root zone and bulk soils. Although none of the species identified appeared to represent foodborne pathogens, several have been linked to clinical implications. For example, *S. marcescens*, which was found in the BS and RZ samples, is sometimes associated with MDR nosocomial infections in hospitals (Mahlen, 2011), and *P. aeruginosa* and *B. cepacia* have both been associated with those affecting pediatric patients (Oberdorfer et al., 2009). Thus, understanding the environmental transmission of soilborne pathogens that can move into healthcare settings, such as via foods, has implications for public health. Several MDR isolates from our study were consistent with those identified on ready-to-eat salads, indicating that AMR may even support persistence after postharvest washing and minimal processing (Zhou et al., 2020). Future research on the mechanistic role of ARGs in transmission chains for food safety warrants investigation.

To characterize the AMR genotypes, we used a complementary approach with two reference tools and databases: AMRFinderPlus and CARD. AMRFinderPlus is used in standard NCBI Pathogen Detection archival characterizations (https://www.ncbi.nlm.nih.gov/pathogens/) and is considered to more stringently find gene variants linked to conferred resistance, though it has a bias towards model and pathogenic organisms (Feldgarden et al., 2021). Alternatively, the CARD database has a more comprehensive reference dataset, though it is less sensitive in its predictions. That is, gene matches with CARD have been described to correlate with AMR resistance mechanisms rather than be an accurate indicator of resistance (e.g., more information on variants is sometimes needed; Skoulakis et al., 2025). As such, we prioritized the ARGs identified by AMRFinderPlus in efforts to understand the observed phenotypes, while also considering the approximately 4-fold larger set of ARGs identified using CARD. Overall, there was a wide variety of ARGs found among the MDR isolates, which were largely conserved at species and genus levels. Despite these similar genotypes, phenotypic variation was still observed within certain taxonomic groups (e.g., *Serratia*, *Providencia*, *Bacillus*), suggesting that alternative regulatory elements or additional genes that were not annotated as AMR genes (e.g., hypothetical proteins) may play potential roles in the observed differences. Future mechanistic studies are essential to validate AMR genotype-phenotype relationships and expand ARG reference databases, which may be biased towards model organisms, to improve genotypic characterization of diverse environmental resistomes.

ARGs play important roles in bacterial survival in highly competitive niches within soil and plant microenvironments. Certain ARGs, such as those within the *van, bla, AAC*, and *tet* gene families, can be traced back to bacteria suspended in permafrost, long predating the isolation and use of antibiotics in medicine (D’Costa et al., 2011). Microbial efflux pumps, which were the most abundant class of ARGs identified among isolates in this study, are essential for modulating chemical stress for survival within soils (Gaurav et al., 2023). We note that efflux mechanisms may have dominated among the MDR isolates in this work due to initial bacterial enumeration/enrichment on Amp and Tet plates, as resistance to these antibiotics, especially Tet, is often conferred by efflux pumps. Additional mechanisms of resistance related to *Streptomyces*-derived antibiotics, such as beta-lactams, glycopeptides, and tetracyclines, play important roles in soil persistence and perhaps on plant surfaces as well (Alam et al., 2022). We also found a variety of biocide and metal tolerance genes in our genomes, including *fieF*, which was associated with plasmids and CE sequences in many genomes. Metal tolerance genes have been found to transfer with ARGs and MGEs in metagenomic studies (Li et al., 2022; Liu et al., 2024). Gene sharing via horizontal transmission further drives ARG diversity in the complex preharvest ecosystem (Shen et al., 2019; Wang et al., 2022). Due to soil being a reservoir for antibiotics and ARGs, it is known that pathogenic organisms often acquire ARGs from environmental organisms via MGEs (Forsberg et al., 2012). Our study identified key gene associations among certain MGEs, primarily plasmids. Further investigating the potential for mobilization and transfers to opportunistic or pathogenic bacteria would have applications for supporting food safety.

Overall, this research provides novel information regarding the types of MDR bacteria found in urban agriculture systems and the specific ARGs, stress response genes, and MGEs that they encode. We recognize potential limitations based on the spatial and temporal scale in our sampling and sequencing approach, along with initial antibiotic selection towards efflux-related resistance (i.e., Amp and Tet). Incorporating additional antibiotics into selective enrichments, adding a metagenomics perspective to cover the broader environmental resistome, and incorporating long-read sequencing into WGS and/or metagenomics workflows would be useful in future studies to better understand the role of MGEs in AMR dissemination. Nevertheless, our findings demonstrate potential universality of AMR in urban agriculture systems and underscore the importance of developing control strategies to enhance food safety.

## Conclusions

This study presents a novel collection of MDR bacteria recovered from soils and preharvest leafy greens at urban farms and community gardens. Using a comprehensive bioinformatics approach, we identified the taxa and genotypes potentially contributing to MDR in these environments. Although the isolates were not identified as foodborne pathogens, some carried ARGs in proximity MGE sequences, primarily plasmid elements. Further validation of the role of plasmids carrying ARGs in the conferred phenotypes, such as with long-read sequencing and mechanistic investigations, would be needed to understand the potential for ARG mobilization. While our findings provide a snapshot of the culturable MDR bacteria present, broader molecular approaches such as using metagenomics would offer deeper insights into the microbiomes and resistomes associated with urban food systems. As urban agriculture continues to expand globally, further investigation into the microbiota associated with these unique environments becomes essential to inform strategies that will ensure food safety.

## Data Availability

The raw sequencing data and genome assemblies presented in the study are deposited in the NCBI Sequence Read Archive and GenBank, respectively, under the BioProject number PRJNA1307595. Scripts and code that may be used to reproduce our analyses are available at: https://github.com/foodmicrobiome/2025_Harrelson_UrbanFarm.

## Supplementary material

Supplementary Figure 1
**(A)** ANI of the two unclassified *Chryseobacterium* species. **(B)** ANI of the two unclassified *Providencia* species.

Supplementary Figure 2 AMR genes identified by CARD and potential associations with MGEs. **(A)**Percent of isolates carrying the top 50 CARD genes, with the colors corresponding to antibiotic class. **(B)** Counts for occurrences of MGEs and AMR genes found within 5,000 bp on the same contig.

Supplementary Table 1 Chi-squared test statistics comparing the fractions of genera **(A)**, antibiotic resistance phenotypes **(B)**, genotypes detected with AMRFinderPlus **(C)**, and genotypes detected with CARD **(D)** across sample types (i.e., LT, RZ, BS). Significant differences (*P* < 0.05) are in shown bold font.

Supplementary Table 2 Genotype, phenotype, and farm metadata for each MDR isolate.

Supplementary Table 3 AMRFinderPlus and CARD matches found within 10 bp of each other.

Supplementary Table 4 AMRFinderPlus and MobileOG-db hits within 5,000 bp of each other.

Supplementary Table 5 CARD and MobileOG-db hits within 5,000 bp of each other.

## References

[B1] Abadias M. Usall J. Anguera M. Solsona C. Viñas I. (2008). Microbiological quality of fresh, minimally-processed fruit and vegetables, and sprouts from retail establishments. Int. J. Food Microbiol. 123, 121–129. doi: 10.1016/j.ijfoodmicro.2007.12.013, PMID: 18237811

[B2] Alam K. Mazumder A. Sikdar S. Zhao Y.-M. Hao J. Song C.. (2022). Streptomyces: The biofactory of secondary metabolites. Front. Microbiol. 13. doi: 10.3389/fmicb.2022.968053, PMID: 36246257 PMC9558229

[B3] Alcock B. P. Huynh W. Chalil R. Smith K. W. Raphenya A. R. Wlodarski M. A.. (2023). CARD 2023: expanded curation, support for machine learning, and resistome prediction at the Comprehensive Antibiotic Resistance Database. Nucleic Acids Res. 51, D690–D699. doi: 10.1093/nar/gkac920, PMID: 36263822 PMC9825576

[B4] Arkin A. P. Cottingham R. W. Henry C. S. Harris N. L. Stevens R. L. Maslov S.. (2018). KBase: the United States department of energy systems biology knowledgebase. Nat. Biotechnol. 36, 566–569. doi: 10.1038/nbt.4163, PMID: 29979655 PMC6870991

[B5] Bolger A. M. Lohse M. Usadel B. (2014). Trimmomatic: a flexible trimmer for Illumina sequence data. Bioinformatics 30, 2114–2120. doi: 10.1093/bioinformatics/btu170, PMID: 24695404 PMC4103590

[B6] Brown C. L. Mullet J. Hindi F. Stoll J. E. Gupta S. Choi M.. (2022). mobileOG-db: a manually curated database of protein families mediating the life cycle of bacterial mobile genetic elements. Appl. Environ. Microbiol. 88, e00991–e00922. doi: 10.1128/aem.00991-22, PMID: 36036594 PMC9499024

[B7] Buchfink B. Xie C. Huson D. H. (2015). Fast and sensitive protein alignment using DIAMOND. Nat. Methods 12, 59–60. doi: 10.1038/nmeth.3176, PMID: 25402007

[B8] Centers for Disease Control and Prevention (U.S.) (2019). Antibiotic resistance threats in the United States 2019 (Centers for Disease Control and Prevention (U.S). doi: 10.15620/cdc:82532

[B9] Chaumeil P.-A. Mussig A. J. Hugenholtz P. Parks D. H. (2022). GTDB-Tk v2: memory friendly classification with the genome taxonomy database. Bioinformatics 38, 5315–5316. doi: 10.1093/bioinformatics/btac672, PMID: 36218463 PMC9710552

[B10] Chen P. Yu K. He Y. (2023). The dynamics and transmission of antibiotic resistance associated with plant microbiomes. Environ. Int. 176, 107986. doi: 10.1016/j.envint.2023.107986, PMID: 37257204

[B11] D’Costa V. M. King C. E. Kalan L. Morar M. Sung W. W. L. Schwarz C.. (2011). Antibiotic resistance is ancient. Nature 477, 457–461. doi: 10.1038/nature10388, PMID: 21881561

[B12] Delgado-Baquerizo M. Eldridge D. J. Liu Y.-R. Sokoya B. Wang J.-T. Hu H.-W.. (2021). Global homogenization of the structure and function in the soil microbiome of urban greenspaces. Sci. Adv. 7, eabg5809. doi: 10.1126/sciadv.abg5809, PMID: 34244148 PMC8270485

[B13] Diaconu E. L. Alba P. Feltrin F. Di Matteo P. Iurescia M. Chelli E.. (2021). Emergence of incHI2 plasmids with mobilized colistin resistance (mcr)-9 gene in ESBL-producing, multidrug-resistant salmonella typhimurium and its monophasic variant ST34 from food-producing animals in Italy. Front. Microbiol. 12. doi: 10.3389/fmicb.2021.705230, PMID: 34335538 PMC8322855

[B14] Doyle M. E. (2015). Multidrug-resistant pathogens in the food supply. Foodborne Pathog. Dis. 12, 261–279. doi: 10.1089/fpd.2014.1865, PMID: 25621383

[B15] Feldgarden M. Brover V. Gonzalez-Escalona N. Frye J. G. Haendiges J. Haft D. H.. (2021). AMRFinderPlus and the Reference Gene Catalog facilitate examination of the genomic links among antimicrobial resistance, stress response, and virulence. Sci. Rep. 11, 12728. doi: 10.1038/s41598-021-91456-0, PMID: 34135355 PMC8208984

[B16] Fiedler G. Kabisch J. Böhnlein C. Huch M. Becker B. Cho G.-S.. (2017). Presence of human pathogens in produce from retail markets in northern Germany. Foodborne Pathog. Dis. 14, 502–509. doi: 10.1089/fpd.2016.2258, PMID: 28594569

[B17] Forsberg K. J. Reyes A. Wang B. Selleck E. M. Sommer M. O. A. Dantas G. (2012). The shared antibiotic resistome of soil bacteria and human pathogens. Science 337, 1107–1111. doi: 10.1126/science.1220761, PMID: 22936781 PMC4070369

[B18] Franklin A. M. Weller D. L. Durso L. M. Bagley M. Davis B. C. Frye J. G.. (2024). A one health approach for monitoring antimicrobial resistance: developing a national freshwater pilot effort. Front. Water 6. doi: 10.3389/frwa.2024.1359109, PMID: 38855419 PMC11157689

[B19] Fu Y. Zhu Y. Dong H. Li J. Zhang W. Shao Y.. (2023). Effects of heavy metals and antibiotics on antibiotic resistance genes and microbial communities in soil. Process Saf. Environ. Prot. 169, 418–427. doi: 10.1016/j.psep.2022.11.020

[B20] Gaurav A. Bakht P. Saini M. Pandey S. Pathania R. (2023). Role of bacterial efflux pumps in antibiotic resistance, virulence, and strategies to discover novel efflux pump inhibitors. Microbiology 169, 001333. doi: 10.1099/mic.0.001333, PMID: 37224055 PMC10268834

[B21] Gu Z. (2022). Complex heatmap visualization. iMeta 1, e43. doi: 10.1002/imt2.43, PMID: 38868715 PMC10989952

[B22] Gu Z. Gu L. Eils R. Schlesner M. Brors B. (2014). *circlize* implements and enhances circular visualization in R. Bioinformatics 30, 2811–2812. doi: 10.1093/bioinformatics/btu393, PMID: 24930139

[B23] Gurmessa B. Pedretti E. F. Cocco S. Cardelli V. Corti G. (2020). Manure anaerobic digestion effects and the role of pre- and post-treatments on veterinary antibiotics and antibiotic resistance genes removal efficiency. Sci. Total Environ. 721, 137532. doi: 10.1016/j.scitotenv.2020.137532, PMID: 32179343

[B24] Kiplimo D. Mwirichia R. Wicaksono W. A. Berg G. Abdelfattah A. (2025). Intrinsic and acquired antimicrobial resistomes in plant microbiomes: implications for agriculture and public health. J. Sust Agri Env. 4, e70049. doi: 10.1002/sae2.70049

[B25] Li X. Rensing C. Vestergaard G. Arumugam M. Nesme J. Gupta S.. (2022). Metagenomic evidence for co-occurrence of antibiotic, biocide and metal resistance genes in pigs. Environ. Int. 158, 106899. doi: 10.1016/j.envint.2021.106899, PMID: 34598063

[B26] Liu Z.-T. Ma R.-A. Zhu D. Konstantinidis K. T. Zhu Y.-G. Zhang S.-Y. (2024). Organic fertilization co-selects genetically linked antibiotic and metal(loid) resistance genes in global soil microbiome. Nat. Commun. 15, 5168. doi: 10.1038/s41467-024-49165-5, PMID: 38886447 PMC11183072

[B27] Mahlen S. D. (2011). Serratia infections: from military experiments to current practice. Clin. Microbiol. Rev. 24, 755–791. doi: 10.1128/CMR.00017-11, PMID: 21976608 PMC3194826

[B28] Naditz A. L. Dzieciol M. Wagner M. Schmitz-Esser S. (2019). Plasmids contribute to food processing environment–associated stress survival in three Listeria monocytogenes ST121, ST8, and ST5 strains. Int. J. Food Microbiol. 299, 39–46. doi: 10.1016/j.ijfoodmicro.2019.03.016, PMID: 30953994

[B29] Nguyen C. C. Hugie C. N. Kile M. L. Navab-Daneshmand T. (2019). Association between heavy metals and antibiotic-resistant human pathogens in environmental reservoirs: A review. Front. Environ. Sci. Eng. 13, 46. doi: 10.1007/s11783-019-1129-0

[B30] Nugent A. Allison S. D. (2022). A framework for soil microbial ecology in urban ecosystems. Ecosphere 13, e3968. doi: 10.1002/ecs2.3968

[B31] Oberdorfer P. Pongwilairat N. Washington C. H. (2009). Nosocomial infections among pediatric patients with neoplastic diseases. Int. J. Pediatr. 2009, 1–5. doi: 10.1155/2009/721320, PMID: 20049342 PMC2798098

[B32] Olm M. R. Brown C. T. Brooks B. Banfield J. F. (2017). dRep: a tool for fast and accurate genomic comparisons that enables improved genome recovery from metagenomes through de-replication. ISME J. 11, 2864–2868. doi: 10.1038/ismej.2017.126, PMID: 28742071 PMC5702732

[B33] Parks D. H. Imelfort M. Skennerton C. T. Hugenholtz P. Tyson G. W. (2015). CheckM: assessing the quality of microbial genomes recovered from isolates, single cells, and metagenomes. Genome Res. 25, 1043–1055. doi: 10.1101/gr.186072.114, PMID: 25977477 PMC4484387

[B34] Prjibelski A. Antipov D. Meleshko D. Lapidus A. Korobeynikov A. (2020). Using SPAdes *de novo* assembler. CP Bioinf. 70, e102. doi: 10.1002/cpbi.102, PMID: 32559359

[B35] Rahman M. Alam M.-U. Luies S. K. Kamal A. Ferdous S. Lin A.. (2021). Contamination of fresh produce with antibiotic-resistant bacteria and associated risks to human health: A scoping review. IJERPH 19, 360. doi: 10.3390/ijerph19010360, PMID: 35010620 PMC8744955

[B36] Samreen Ahmad I. Malak H. A. Abulreesh H. H. (2021). Environmental antimicrobial resistance and its drivers: a potential threat to public health. J. Global Antimicrobial Resistance 27, 101–111. doi: 10.1016/j.jgar.2021.08.001, PMID: 34454098

[B37] Scallan E. Hoekstra R. M. Angulo F. J. Tauxe R. V. Widdowson M.-A. Roy S. L.. (2011). Foodborne illness acquired in the United States—Major pathogens. Emerg. Infect. Dis. 17, 7–15. doi: 10.3201/eid1701.P11101, PMID: 21192848 PMC3375761

[B38] Schoen M. Jahne M. Garland J. (2024). Semi-Systematic Literature Review: Impacts of Antibiotic Pesticides on Antibiotic Resistance in Human and Animal Pathogens and Commensals to Inform Risk Assessment (Washington, DC: U.S. Environmental Protection Agency).

[B39] Shen Y. Stedtfeld R. D. Guo X. Bhalsod G. D. Jeon S. Tiedje J. M.. (2019). Pharmaceutical exposure changed antibiotic resistance genes and bacterial communities in soil-surface- and overhead-irrigated greenhouse lettuce. Environ. Int. 131, 105031. doi: 10.1016/j.envint.2019.105031, PMID: 31336252

[B40] Sievert C. (2020). Interactive Web-Based Data Visualization with R, plotly, and shiny (Chapman and Hall/CRC). Available online at: https://plotly-r.com (Accessed May 1, 2025).

[B41] Skoulakis A. Kostoula V. Daniilidis K. Gkountinoudis C.-G. Petinaki E. Hatzigeorgiou A. G. (2025). AmrProfiler: a comprehensive tool for identifying antimicrobial resistance genes and mutations across species. Nucleic Acids 53, W20–W31. doi: 10.1093/nar/gkaf400, PMID: 40347103 PMC12230725

[B42] Sutton K. F. Ashley L. W. (2024). Antimicrobial resistance in the United States: Origins and future directions. Epidemiol. Infect. 152, e33. doi: 10.1017/S0950268824000244, PMID: 38343135 PMC10894903

[B43] Walter E. J. S. Cui Z. Tierney R. Griffin P. M. Hoekstra R. M. Payne D. C.. (2025). Foodborne illness acquired in the United States—Major pathogens 2019. Emerg. Infect. Dis. 31. doi: 10.3201/eid3104.240913, PMID: 40133035 PMC11950263

[B44] Wang X. Lan B. Fei H. Wang S. Zhu G. (2021). Heavy metal could drive co-selection of antibiotic resistance in terrestrial subsurface soils. J. Hazardous Materials 411, 124848. doi: 10.1016/j.jhazmat.2020.124848, PMID: 33858075

[B45] Wang H. Qi J.-F. Qin R. Ding K. Graham D. W. Zhu Y.-G. (2023). Intensified livestock farming increases antibiotic resistance genotypes and phenotypes in animal feces. Commun. Earth Environ. 4, 123. doi: 10.1038/s43247-023-00790-w

[B46] Wang F. Sun R. Hu H. Duan G. Meng L. Qiao M. (2022). The overlap of soil and vegetable microbes drives the transfer of antibiotic resistance genes from manure-amended soil to vegetables. Sci. Total Environ. 828, 154463. doi: 10.1016/j.scitotenv.2022.154463, PMID: 35276164

[B47] Wickham H. (2016). ggplot2: Elegant Graphics for Data Analysis (Springer-Verlag New York). Available online at: https://ggplot2.tidyverse.org (Accessed May 1, 2025).

[B48] Yang J.-L. Zhang G.-L. (2015). Formation, characteristics and eco-environmental implications of urban soils – A review. Soil Sci. Plant Nutr. 61, 30–46. doi: 10.1080/00380768.2015.1035622

[B49] Zeng Q. Lam K. Salcedo A. Tikekar R. V. Micallef S. A. Blaustein R. A. (2024). Effects of organic soil amendments on antimicrobial-resistant bacteria in urban agriculture environments. J. Food Prot. 87, 100344. doi: 10.1016/j.jfp.2024.100344, PMID: 39147100

[B50] Zhang Y.-J. Hu H.-W. Chen Q.-L. Singh B. K. Yan H. Chen D.. (2019). Transfer of antibiotic resistance from manure-amended soils to vegetable microbiomes. Environ. Int. 130, 104912. doi: 10.1016/j.envint.2019.104912, PMID: 31220751

[B51] Zhou S.-Y.-D. Wei M.-Y. Giles M. Neilson R. Zheng F. Zhang Q.. (2020). Prevalence of antibiotic resistome in ready-to-eat salad. Front. Public Health 8. doi: 10.3389/fpubh.2020.00092, PMID: 32269985 PMC7109403

