# Supplementary figures and images for: Multidrug resistance in bacteria associated with leafy greens and soil in urban agriculture systems
Source: Front Plant Sci. 2025 Sep 22;16:1664284. doi: 10.3389/fpls.2025.1664284 (PMC12497839; doi:10.3389/fpls.2025.1664284)

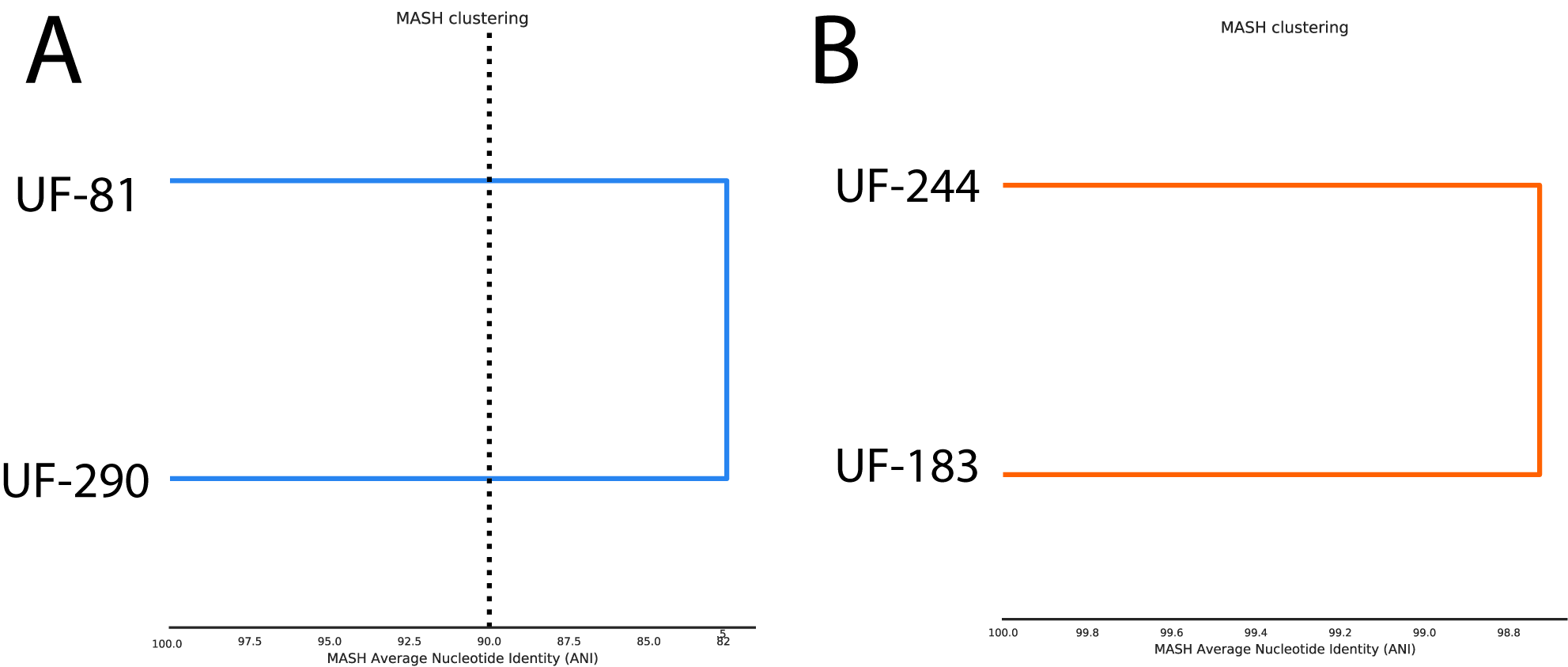

Supplement: Supplementary Figure 1 — (A) ANI of the two unclassified Chryseobacterium species. (B) ANI of the two unclassified Providencia species. [file Image1.tif]

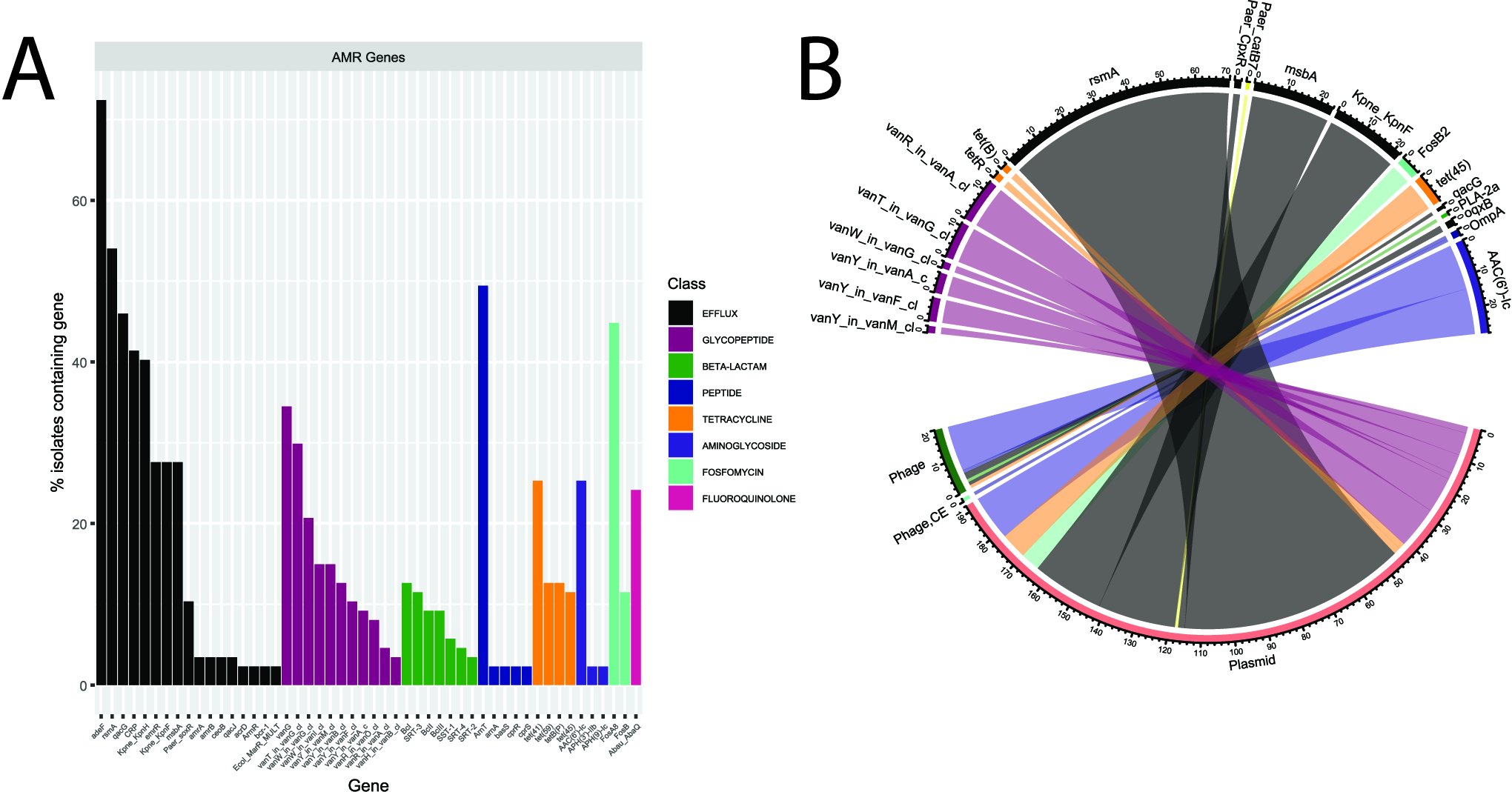

Supplement: Supplementary Figure 2 — AMR genes identified by CARD and potential associations with MGEs. (A)Percent of isolates carrying the top 50 CARD genes, with the colors corresponding to antibiotic class. (B) Counts for occurrences of MGEs and AMR genes found within 5,000 bp on the same contig. [file Image2.tif]
